# A New Method for Quick and Easy Hemolymph Collection from Apidae Adults

**DOI:** 10.1371/journal.pone.0170487

**Published:** 2017-01-26

**Authors:** Grzegorz Borsuk, Aneta A. Ptaszyńska, Krzysztof Olszewski, Marcin Domaciuk, Patcharin Krutmuang, Jerzy Paleolog

**Affiliations:** 1 Department of Biological Basis of Animal Production, Faculty of Biology, Animal Sciences and Bioeconomy, University of Life Sciences, Lublin, Poland; 2 Department of Botany and Mycology, Institute of Biology and Biochemistry, Faculty of Biology and Biotechnology, Maria Curie-Skłodowska University, Lublin, Poland; 3 Department of Plant Anatomy and Cytology, Institute of Biology and Biochemistry, Faculty of Biology and Biotechnology, Maria Curie-Skłodowska University, Lublin, Poland; 4 Department of Entomology and Plant Pathology, Faculty of Agriculture, Chang Mai University, Chiang Mai, Thailand; 5 Department of Zoology, Animal Ecology & Wildlife Management, Faculty of Biology, Animal Sciences and Bioeconomy, University of Life Sciences in Lublin, Lublin, Poland; Colorado State University, UNITED STATES

## Abstract

Bio-analysis of insects is increasingly dependent on highly sensitive methods that require high quality biological material, such as hemolymph. However, it is difficult to collect fresh and uncontaminated hemolymph from adult bees since they are very active and have the potential to sting, and because hemolymph is rapidly melanized. Here we aimed to develop and test a quick and easy method for sterile and contamination-free hemolymph sampling from adult Apidae. Our novel antennae method for hemolymph sampling (AMHS), entailed the detachment of an antenna, followed by application of delicate pressure to the bee's abdomen. This resulted in the appearance of a drop of hemolymph at the base of the detached antenna, which was then aspirated using an automatic pipetter. Larger insect size corresponded to easier and faster hemolymph sampling, and to a greater sample volume. We obtained 80–100 μL of sterile non-melanized hemolymph in 1 minute from one *Bombus terrestris* worker, in 6 minutes from 10 *Apis mellifera* workers, and in 15 minutes from 18 *Apis cerana* workers (+/−0.5 minutes). Compared to the most popular method of hemolymph collection, in which hemolymph is sampled by puncturing the dorsal sinus of the thorax with a capillary (TCHS), significantly fewer bees were required to collect 80–100 μL hemolymph using our novel AMHS method. Moreover, the time required for hemolymph collection was significantly shorter using the AMHS compared to the TCHS, which protects the acquired hemolymph against melanization, thus providing the highest quality material for biological analysis.

## Introduction

Apidological research is increasingly reliant on modern highly sensitive analytical methods. Obtaining credible results with these techniques requires properly sampled, well-prepared, and correctly preserved biological material. In particular, hemolymph studies are needed to investigate insect physiological states. However, the collection of hemolymph from Apidae adults is difficult.

Hemolymph from adult bees has been analyzed in immunological, nutritional, mycological, proteomic, epigenetic, and biochemical research [1–5]. Methods for collecting apian hemolymph include decapitation, cutting the tibia or femur, puncturing the heart, puncturing the dorsal or ventral sinus of the thorax, or puncturing the dorsal aorta between the head and thorax [3,6,7]. However, these sampling methods require specialized equipment, such as glass micro-capillaries, specially prepared plastic Pasteur pipettes [1,3,6]. Mayack and Naug [8] describe a method, hemolymph is acquired by clipping bee antennae and then, and spinning bees in centrifuge tubes at 16,000 RCF for 30 s to obtain hemolymph. The use of such tools carries a risk of contaminating the samples with crop or intestinal contents during collection or increase risk of melanization by extending its processing time. The collection of hemolymph from adult insects is further complicated by these organisms’ propensity for movement and their ability to sting the collector. The above-cited studies include descriptions of methods, but do not report any quantitative characteristics of these methods. The “ideal method” for hemolymph sampling is controversial [9], and this still remains a problem “of the general interest” within entomological studies [9].

In our present study, we aimed to develop and test a new, quick, and relatively easy method for sterile and contamination-free collection of hemolymph from Apidae adults. Our novel method involves sampling from Apidae adult antennae, which we compared to the most popular method of hemolymph sampling by puncturing the dorsal sinus of the thorax with a capillary.

## Materials and Methods

### Novel antennae method of hemolymph sampling (AMHS)

Our novel antennae method of hemolymph sampling (AMHS) required the following equipment: a gas burner, a rectangular Styrofoam plate for holding an insect, tweezers, an automatic pipetter, pipette tips, 70% ethanol, cotton swabs, and an ice-bath to cool 0.2-mL Eppendorf tubes. The equipment and laboratory room were sterilized with a UV lamp. The procedure was performed in the immediate vicinity of a working gas burner to maintain sterile air.

**Fig 1:**
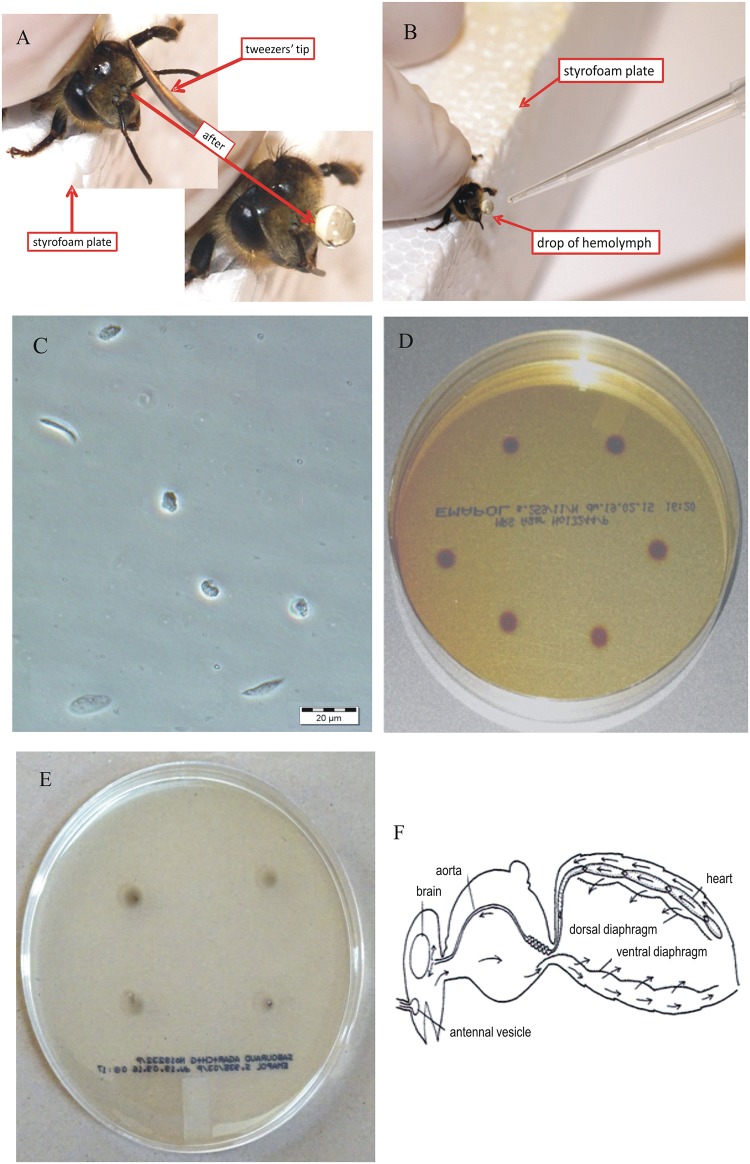
New antenna method of hemolymph collection. (A) Method for detaching an antenna and inducing hemolymph outflow in a worker honeybee. (B) Collection of hemolymph into a pipette. (C) Microphotograph of hemolymph sampled with the new antennae method (Olympus BX 6; magnification, 500×). (D) Hemolymph samples placed on MRS medium to confirm their sterility (E) Hemolymph samples placed on Sabouraud medium to prove their sterility. (F) Hemolymph circulation in a bee’s body [11].

For this method, the researcher used the index finger and thumb of the non-dominant hand to hold the bee by its thorax. The bee was placed with its thorax gently pressed against the edge of a Styrofoam plate (Fig 1A and 1B), a position that prevented the bee from stinging the researcher’s fingers. Using the dominant hand, the researcher dipped a cotton swab in 70% ethanol and used it to disinfect the area around the bee’s antennae (this step is not required if hemolymph is being collected for non-microbiological studies). Following ethanol evaporation, the researcher used the dominant hand to grasp the tip of an antenna with tweezers, and to detach the antenna from its base with an energetic movement. Next, the tweezers were put away and the researcher picked up a pipetter with the dominant hand. With the index finger of the non-dominant hand, the researcher gently pressed the bee’s abdomen, increasing hemolymph pressure, and leading to the appearance of a drop of pure hemolymph (Fig 1A). This drop was immediately transferred with the automatic pipetter (Fig 1B) into an Eppendorf tube, and placed in an ice-bath (2–4°C) to prevent melanization. Hemolymph sampled in this manner, could be immediately analyzed or frozen at −40°C for future studies (storage at −80°C is recommended if the storage period is to be longer than one month).

### Standard method of puncturing the dorsal sinus of the thorax with capillary of hemolymph sampling (TCHS)

For comparison with our new AMHS method, we also sampled hemolymph using the popular method of puncturing the dorsal sinus of the thorax (TCHS). The TCHS method required the same equipment as specified above for AMHS, as well as glass capillaries with a thin sharp end, and a micro-pump to push hemolymph from the capillaries to Eppendorf tubes. In this method, bees were held by the head and thorax. The bee abdomen was laid on a Styrofoam plate, and the capillary was used to pierce abdomen segments between termites 3 and 4. Hemolymph was taken into the capillary, and then a micro-pump was used to push this hemolymph from the capillary into an Eppendorf tube.

### Biological material used in the AMHS and TCHS methods

Immediately after emergence, *Apis mellifera carnica* workers and drones were individually marked with a paint marker and left inside their hive. Upon reaching 10 days of age, 80 marked workers and 80 marked drones were captured, placed in two separate cages, and transported to the laboratory for hemolymph sampling. The groups of drones and workers were each divided into two halves, such that 40 individuals of each type were used for AMHS and TCHS. Hemolymph collection was performed immediately after the bees were delivered to the laboratory, no more than 30 minutes after their removal from their hive.

We also removed 40 individually caged virgin 4-day-old honeybee queens, each accompanied by five workers, from a colony lacking a nurse queen. Hemolymph was collected from these queens within 30 minutes of their removal from the colony. Of the 40 hemolymph samples taken from the queens, 20 were extracted by AMHS and 20 by TCHS.

80 *Bombus terrestris* worker bees were captured while flying outside their nest entrance. These bees were placed in a cage, and used for hemolymph sampling within 30 minutes after their capture. Of these 80 worker bees, 40 were used for AMHS and 40 for TCHS. All procedures (*A*. *mellifera*, *B*. *terestris*) were performed at the University of Life Sciences, in Lublin, Poland (51°13′ N, 22°38′ E).

From the apiary of Chang Mai University, Thailand (18°48'21.4"N; 98°57'39.4"E), 80 *Apis cerana* worker bees were captured, and used for hemolymph collection: 40 by AMHS and 40 by TCHS.

The cages, and caged bee management applied in honeybees, Cerana bees and bumblebees, have been previously described in details by Borsuk et al. [10].

### Volume and quality evaluation of hemolymph samples obtained using the AMHS and TCHS methods

In both AMHS and TCHS, we assessed total hemolymph volumes by pooling the hemolymph taken from multiple bees in a single Eppendorf tube, and then determining the total amount of hemolymph using an automatic pipette. Although hemolymph volume could have been determined using a pipette scale during collection in the AMHS method, we chose to utilize the same measurement method for both techniques to improve the validity of comparison. We also measured the total time that it took to collect 80–100 μL of hemolymph in an Eppendorf tube.

To confirm that the fluid collected by AMHS was pure hemolymph, we sampled hemolymph from six insects of each of the studied bee species and castes. From these samples, microscopic hemolymph slides were prepared, and examined for the presence of properly developed normal hemocytes, and to detect any other possible contaminations, like pollen grains, etc. To check hemolymph sterility, we used two different agar mediums: MRS to detect bacterial growth and Sabouraud to assess fungal growth. From individuals of each studied bee species and caste (Table 1), five drops of 5 μL of hemolymph obtained by AMHS were placed on both MRS and Sabouraud agar plates. In total, 100 hemolymph samples were placed on 20 MRS and 20 Sabouraud agar plates. Microorganism growth was detected after 72 hours of incubation at 37°C for MRS, or and 30°C for Sabouraud.

**Table 1:** Characteristics of the new antennae method for hemolymph sampling (AMHS) versus the common method of puncturing the dorsal sinus of the thorax with capillary for hemolymph sampling (TCHS).

Bee species and caste		Age (days)	Number of bees		Number of individuals needed to obtain 80–100 μl of hemolymph				Sampling time needed to obtain 80–100 μl of hemolymph (+/- 0.5 mins)			
			AMHS	TCHS	AMHS		TCHS		AMHS		TCHS	
					Mean	SD	Mean	SD	Mean	SD	Mean	SD
*Apis mellifera carnica*	workers	10	40	40	10^A^	0.20	40^B^	0.42	6^A^	0.20	15^B^	0.12
	drones	10	40	40	2^a^	0.13	5^b^	0.18	1^A^	0.12	6^B^	0.18
	queens	4	20	20	3^a^	0.15	6^b^	0.19	2^A^	0.11	8^B^	0.17
*Apis cerana*	workers	10	40	40	18^a^	0.12	38^b^	0.34	15^A^	0.13	40^B^	0.38
*Bombus terrestris*	workers	-	40	40	1^a^	0.11	3^b^	0.17	1^A^	0.12	6^B^	0.20

^A, B^: Capital letters indicate that differences between TCHS and AMHS characteristics are significant at the level of p<0.01 (ANOVA).

^a, b^: Lowercase letters indicate that differences between TCHS and AMHS characteristics are significant at the level of p<0.05 (ANOVA).

SD: Standard deviation.

Finally, we compared the workload of the AMHS to that of the frequently used TCHS. We evaluated the time and the number of adult bees required to collect 80–100 μL of hemolymph. The personnel of our laboratory were familiar with both of these methods, enabling an objective comparison of quantitative characteristics of each method.

### Statistical analysis

Statistical analysis was performed using SAS software version 9.5 (Statistical Analysis System Institute, Cary, NC,). Comparisons between AMHS and TCHS were performed using one-way ANOVA.

## Results and Discussion

Our novel AMHS method is facilitated by the anatomical/functional structure of the bee circulatory system. Circulation is forced by pulsatory contraction of the dorsal and ventral diaphragms, which pump hemolymph into the heart. Next, the aorta transports hemolymph into the head (Fig 1F) [11], supplying hemolymph to the bee’s brain and antennae.

Compared to the TCHS, AMHS enabled the collection of 80–100 μL of sterile hemolymph within a shorter time and from a smaller number of individuals, which minimizing risk of melanization. We believe that during hemolymph collection, it is important to minimize the time of its contact with air. Melanization risk is reduced with a shorter collection time and more rapid progression to the cooling step. In addition to the shortened collection time, the reduced use of insects is another substantial advantage of the AMHS over TCHS. Apidae research should always be designed to minimize the number of bees that must be harmed. The use of AMHS to reduce the number of bees needed to collect a required hemolymph volume is consistent with the guiding principles for more ethical use of animals in research [12].

Our present results also showed that larger insect size was associated with easier and faster hemolymph sampling, and with greater volumes of collected hemolymph (Table 1). Hemolymph sampling from *Apis mellifera carnica* and *Apis cerana* workers took longer and required more insects than hemolymph sampling from bumble bees, as the former species are considerably smaller and have less hemolymph.

Hemolymph is most often acquired with capillaries using varying methods, for example, by puncturing the heart, the dorsal or ventral thorax sinus, or the dorsal aorta [1,3,6]. Each of these procedures carries the risk that the ventriculus will be punctured, leading to sample contamination with intestine contents. Such contamination not only renders the sample unusable, but also requires that the capillary must be replaced with a new one or cleaned and disinfected before the next use—thus, increasing the overall hemolymph collection time. Hemolymph sterility can also be compromised by puncturing the membrane connecting abdomen segments. Use of the AMHS eliminates these capillary-related problems. Moreover, hemolymph collection by decapitation [6] may contaminate the hemolymph with nectar leaking from the bee’s stomach. Our novel AMHS resulted in hemolymph samples of high biological quality. Hemocytes were clearly visible in each hemolymph sample acquired by AMHS (Fig 1C). Analysis of each microscope slide revealed that the AMHS-obtained hemolymph samples were pure and transparent, with no contamination (e.g., pollen grains).

We expected that in the performance of AMHS, ethanol disinfection and limited contact between the hemolymph drop and the bee body would ensure sample sterility. This expectation was confirmed by microbiological assays. The incubation of hemolymph acquired by AMHS on both MRS and Sabouraud agar mediums confirmed the hemolymph sterility. No microorganism growth was observed on any of the plated 100 hemolymph drops. Therefore, no quantitative results are presented here. After terminating the incubation period, we only observed dark brown circles of melanized hemolymph around each drop. This melanization occurred during the incubation period, not during the sampling (Fig 1D and 1E). Notably, when the collected hemolymph is not to be used for microbiological testing, the disinfection step can be omitted. It is possible that ethanol applied at the antennal area can affect enzyme or hormone activities.

Factors other than methodology can influence hemolymph collection efficiency. In our previous studies [13,14], we observed that the amount of hemolymph collected from a bee depends not only on the bee caste and age but also on its degree of hydration measured based on the volume of nectar or sugar syrup consumed prior to hemolymph sampling. Ptaszyńska et al. [13] report that sampling of the proper hemolymph volumes is less effective from older bees. Moreover, sampling efficiency may depend on the researcher’s laboratory skills. These factors make it difficult to compare the quantitative characteristics of different hemolymph-sampling protocols among studies performed in different labs and using different organisms. Notably, in our previous investigations, we have sampled hemolymph from thousands of bees using AMHS, as well as other methods, including TCHS [13,14]. Our laboratory personnel have substantial experience in collecting hemolymph from bees of different ages and castes. This experience helped us to compare the different sampling methods, since our findings were not affected by errors due to variable skills of laboratory staff. Our present results indicated that the AMHS was an efficient, clean, and highly accurate method (Table 1) that was less time-consuming than TCHS [1,3,6].

Hemolymph sampling using methods other than the AMHS typically require additional equipment, such as micro-capillary tubes. Moreover, the removal of hemolymph from micro-capillary tubes requires either the use of a special pump, or crushing the tubes and centrifuging them with beads. Another advantage of the AMHS is the potential to use a pipette scale to measure the hemolymph volume collected from any single bee. This is particularly useful when performing biochemical analysis of micro-samples, e.g., for determining enzyme activities [15]. It is worth of noting that when applying methods other than AMHS [1,3,6], pure hemolymph volume can be calculated indirectly, based on the volume of hemolymph thinner in a tube before sampling and the measured volume of hemolymph thinner after sampling. However, such a method can lead to additional error; therefore, some researchers use Hamilton syringes [16]. The AMHS avoids the expense and additional work (e.g., syringe de-clogging, disinfection, etc.) of using Hamilton syringes.

Mayack and Naug [8] method (hemolymph is acquired by spinning bees with clipped antennae) is similar to the AMHS. However, their method requires both a laboratory centrifuge, and the apian mouth parts had to be glued shut to prevent any possible contamination. Moreover, in their study, they dissected the apian guts to assess *Nosema ceranae* infection before spinning the bees. Without this step, the guts would be a likely source of contamination. Their method involve placing bees in centrifuge tubes, and spinning them at 16,000 RCF for 30 s to obtain hemolymph. This process could stratify the hemolymph components (e.g., proteins and hemocytes) and carries increased risks of microbiological contamination and of hemolymph melanization (see discussion above). While it may be ideal for analyzing hemolymph sugar levels among the honeybee foragers with previously removed guts (as in their study), it is not entirely appropriate for microbiological and genetic studies or investigations of enzyme activities. In this context, our new AMHS method is more suitable for general use, being easy and simple, and likely applicable in a wide range of different types of studies.

## Conclusions

Our novel method for hemolymph collection has many advantages over traditional methods. By minimizing the procedural steps and by not requiring that the bee’s body be punctured, the AMHS decreases the risks of hemolymph contamination. Moreover, the AMHS minimizes the risk of hemolymph melanization, since the hemolymph is rapidly collected into the pipette, minimizing the contact with air. Importantly, the AMHS allows accurate measurement of the hemolymph volume collected from an bee during sampling, which is crucial for many biochemical tests. By maximizing the hemolymph volume obtainable from each bee, the AMHS minimizes the number of bee specimens necessary to collect a required volume of hemolymph. Thus, this method is very efficient and fast, as well as consistent with the ethical principles of biological research. Moreover, the AMHS does not require excessively sophisticated equipment or highly trained staff. The hemolymph collected by AMHS can be processed and analyzed undiluted or with the addition of various buffers and dilatants, and can be analyzed immediately after collection or frozen and stored for later analysis.

Consequently, we believe that the AMHS is an efficient and easy method for rapid collection of large amounts of pure and uncontaminated hemolymph, which can be useful in a wide range of Apidae studies. Moreover, the AMHS can be applied to many insects that have antennae and are similar in size to bees or larger. While we do not have sufficient evidence to claim that AMHS is the best method for all types of entomological research, it undoubtedly seems to be a highly useful technique with few drawbacks.

## References

[1] Chan QW, Howes CG, Foster LJ. Quantitative comparison of caste differences in honeybee hemolymph. Mol Cell Proteomics. 2006; 5: 2252–2262. 10.1074/mcp.M600197-MCP200 16920818

[2] Antúnez K, Martin-Hernandez R, Prieto L, Meana A, Zunino P, Higes M. Immune suppression in the honey bee (*Apis mellifera*) following infection by *Nosema ceranae* (Microsporidia). Environ Microb. 2009; 11: 2284–2290. 10.1111/j.1462-2920.2009.01953.x 19737304

[3] Garido MP, Martin ML, Negri P, Martin EJ. A standardized method to extract and store hemolymph from *Apis mellifera* and the ectoparasites *Varroa destructor* from protein analysis. J Apicult Res. 2013; 52: 67–68.

[4] Strachecka A, Krauze M, Olszewski K, Borsuk G, Paleolog J, Merska M, Chobotow J, Bajda M, Grzywnowicz K. Unexpectedly strong effect of caffeine on the vitality of western honeybees (*Apis mellifera*). Biochemistry. 2014; 79: 1464–1475. 10.1134/S0006297914110066 25540004

[5] Strachecka A, Olszewski K, Krauze M, Paleolog J, Borsuk G, Merska M, Bajda M, Chobotow J. Coenzyme Q10 treatments influence the lifespan and key biochemical resistance systems in the honeybee, Apis mellifera. Arch Ins Biochemi Physiol. 2014; 86: 165–179. 10.1002/arch.21159 24659567

[6] Kostecki R. Investigation on the haemocytes and hemolymph of honeybees. J Apicult Res. 1965; 4: 49–54.

[7] Szymaś B, Jędruszczuk A. The influence of different diets on haemocytes of adult worker honey bees, *Apis mellifera*. Apidologie. 2003; 34: 97–102.

[8] Mayack C, Naug D. Parasitic infection leads to decline in hemolymph sugar levels in honeybee foragers. J Insect Physiol 2010; 56: 1572–1575. 10.1016/j.jinsphys.2010.05.016 20685210

[9] Kobmoo N. How can we extract the hemolymph from insects, especially from small ones like ants? ResearchGate 2012; 8: 19 Available from: https://www.researchgate.net/post/How_can_we_extract_the_hemolymph_from_insects_especially_from_small_ones_like_ants15

[10] Borsuk G, Strachecka A, Olszewski K, Paleolog J. The interaction of worker bees which have increased genotype variance. Part 2. Cage tests of sugar syrup collecting and mortality. J Apicult Sci. 2011; 55: 53–58.

[11] Dade HA. Anatomy and dissection of the honeybee. International Bee Research Association 2009; 70–72.

[12] Franco N, Olsson A. Scientists and the 3Rs: attitudes to animal use in biomedical research and the effect of mandatory training in laboratory animal science. Lab Anim. 2014; 48: 50–60. 10.1177/0023677213498717 23940123

[13] Ptaszyńska AA, Borsuk G, Zdybicka-Barabas A, Cytryńska M, Małek W. Are commercial probiotics and prebiotics effective in the treatment and prevention of honeybee nosemosis C? Parasitol Res. 2015; 115: 397–406. 10.1007/s00436-015-4761-z 26437644 PMC4700093

[14] Ptaszyńska AA, Borsuk G, Mułenko W, Wilk J. Impact of vertebrate probiotics on honeybee yeast microbiota and on the course of nosemosis. Med Weter. 2016; 72: 430–434.

[15] Laughton AM, Siva—Jothy M T. A standardised protocol for measuring phenoloxidase and prophenoloxidase in the honey bee, Apis mellifera. Apidologie. 2011; 42:140–149.

[16] Mikulecky M, Bounias M. Worker honeybee hemolymph lipid composition and synodic lunar cycle periodicities. Braz J Med Biol Res. 1997; 30: 275–279. 9239316 10.1590/s0100-879x1997000200018

